# The decrease of non-complicated acute appendicitis and the negative appendectomy rate during pandemic

**DOI:** 10.1007/s00068-021-01663-7

**Published:** 2021-04-12

**Authors:** Marco Ceresoli, Federico Coccolini, Stefano Magnone, Alessandro Lucianetti, Pietro Bisagni, Teodora Armao, Luca Ansaloni, Mauro Zago, Massimo Chiarugi, Fausto Catena, Marco Braga, the Appendicitis-COVID study group, Marco Nizzardo, Luca Nespoli, Luca Fattori, Luca Degrate, Stefano Perrone, Marco Cereda, Michele Pisano, Elia Poiasina, Paolo Bertoli, Michele Ballabio, Stefano Braga, Giorgio Graziano, Dario Tartaglia, Francesco Arces, Marco Mariani, Fulvio Tagliabue, Gennaro Perrone, Alfredo Annicchiarico, Mario Giuffrida, Giovanni Ferrari, Antonio Benedetti, Niccolò Allievi, Michele Ciocca, Enrico Pinotti, Mauro Montuori, Michele Carlucci, Valentina Tomajer, Paola Fugazzola

**Affiliations:** 1 General and Emergency Surgery Dept, School of Medicine and Surgery, Milano-Bicocca University, Via Pergolesi 33, 20900 Monza, Italy; 2 Emergency Surgery and Trauma Center Dept, Pisa University Hospital, Pisa, Italy; 3 General and Emergency Surgery Dept, ASST Papa Giovanni XXIII, Bergamo, Italy; 4 General and Emergency Surgery Dept, ASST Lodi, Lodi, Italy; 5 General and Emergency Surgery Dept, IRCCS San Matteo, University of Pavia, Pavia, Italy; 6 Robotic and Emergency Surgery Dept, ASST Lecco, Ospedale Manzoni, Lecco, Italy; 7 Emergency Surgery Dept, Parma University Hospital, Parma, Italy

**Keywords:** Acute appendicitis, COVID-19, Non-complicated acute appendicitis

## Abstract

**Background:**

During pandemic, admissions for surgical emergencies dropped down dramatically. Also acute appendicitis decreased. The aim of the present study was to evaluate the change in volume and clinical presentation of patients with acute appendicitis during pandemic and the variation in treatment.

**Methods:**

This is a retrospective study of patients admitted in 11 Italian hospital for acute appendicitis during the lockdown period (March–April 2020) compared with the same period of the previous 2 years (2018–2019). The number and the rate of complicated and non-complicated acute appendicitis were recorded and compared between the two study periods; non-operative vs operative treatment and negative appendectomy rate were also recorded.

**Results:**

The study included 532 patients, 112 in the study period and 420 in the control period; Hospital admission for acute appendicitis dropped by 46% (OR 0.516 95% CI 0.411–0.648 *p* < 0.001) during the 2020 lockdown. The number of complicated acute appendicitis did not change (− 18%, OR 0.763 95% CI 0.517–1.124 *p* = 0.1719), whereas the number of non-complicated acute appendicitis significantly decreased (− 56%, OR 0.424 95% CI 0.319–0.564 *p* < 0.001). Non-operative treatment rate remained similar (12.1% vs. 11.6% *p* = 0.434). The negative appendectomy rate also significantly decreased (6.1% vs. 17.3%, *p* = 0.006).

**Conclusions:**

The present study found a significant reduction of both admissions for non-complicated acute appendicitis and negative appendectomy rate during the pandemic period. Conversely, admissions for complicated acute appendicitis did not change.

*Trial registration*: NCT04649996.

## Introduction

Although acute appendicitis is a common cause of hospital admission, its best treatment is still a matter of debate [1]. Advances in diagnostics and the development of clinical scores led to a decline of appendectomy for acute appendicitis [2, 3]. Along with the decrease of the incidence of diagnosis of acute appendicitis, non-operative treatment has been proposed in non-complicated clinical presentation, as reported by Andersson [4, 5].

Appendectomy is burdened with short-term complications, delayed return to work, and long-term morbidity as intestinal obstruction. The negative appendectomy rate remains high, with a considerable proportion of patients with no inflammation at histological examination [6–8]. On the other hand, non-operative treatment is associated with a high recurrence rate [9, 10]. Evidence failed to demonstrate non-operative treatment's superiority, and guidelines recommend it as an alternative option for selected cases [11–14].

To limit the pandemic's rapid spread, there was a generalized lockdown in Northern Italy between March and April 2020: Hospitals had to face an extremely high number of patients with SARS-CoV2 infection, thus requiring a massive re-allocation of resources. Elective surgical activity and hospital admissions for surgical emergencies dropped down, including patients with acute appendicitis [15–19].

The present study aimed to analyze both the volume and characteristics of patients hospitalized for acute appendicitis during the 2-month pandemic lockdown compared with the same period of the previous 2 years.

## Methods

**Fig. 1:**
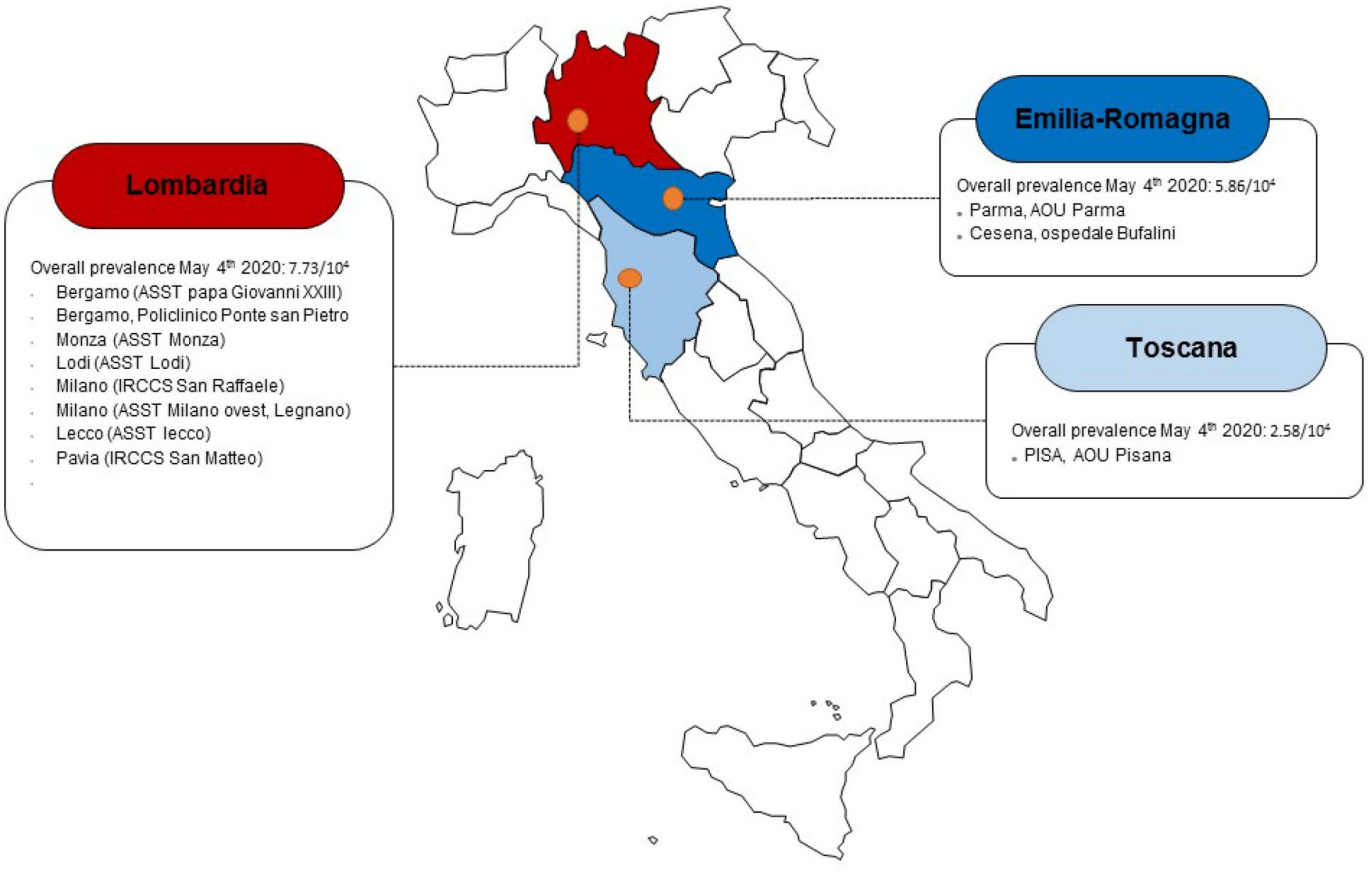
Included study centers with local prevalence of SARS-CoV2 infections

This is a multicentre retrospective study including all patients of any age admitted to 11 Italian referral hospitals for acute appendicitis during the lockdown period, between March 1 and April 30, 2020 (study group) (Fig. 1). Controls were patients admitted to the same hospitals for acute appendicitis during the same 2-month period in 2018 and 2019 (control group). Patients with a diagnosis other than acute appendicitis or appendectomy during other surgeries were excluded.

In each patient demographics, anthropometric data, the Charlson's comorbidity index [20], routine laboratory tests, operative variables, and pathology report were collected.

Acute appendicitis was defined as complicated in case of perforation, abscess, or diffuse peritonitis. Negative appendectomy was considered in case of no acute inflammation at histologic assessment. Postoperative complications were graded according to the Clavien–Dindo classification [21]. Hospital readmissions within 30 days were also recorded.

Categorical variables were shown as a percentage and were compared with the Chi-square test. The distribution of continuous variables was tested with the Kolmogorov–Smirnov test, and data were shown as median and interquartile range (IQR). They were compared with the Mann–Whitney U test as appropriate. The number of cases observed was compared with the Poisson log-linear model and were expressed as variation and Odd Ration (OR) with 95% CI.

The number of confirmed cases of SARS-CoV2 infection, which occurred in the province of each hospital during the study period, was recorded [22]. The percent change of admissions for acute appendicitis comparing the study and the control period was calculated for each hospital and then correlated in a scatter plot with the prevalence of SARS-CoV2 infection expressed as cases/1000 inhabitants. The strength of the resulting correlation was tested with the Pearson test.

The study protocol was registered at clinicaltrials.gov (NCT04649996), and the Ethical Committee approval was obtained. The study was conducted according to the STROBE guidelines [23].

## Results

A total of 532 patients were included in the analysis: 112 during the study period and 420 during the control period. A 46% decrease in hospital admission for acute appendicitis was observed (OR 0.516 95% CI 0.411–0.648 *p* < 0.001).

Table 1 shows that study and control groups were homogeneous except for a higher proportion of men (68.7% vs. 55.0% *p* = 0.009) and of subjects who underwent CT scan (36.1% vs. 19.3%, *p* < 0.001) in the study group.

**Table 1:** Patients’ characteristics

	Control period (2018–2019)				Study period (2020)				
	420				112				
	N	%	Median	IQR	N	%	Median	IQR	
Men	231	55			77	68.75			0.009
Age			29	18–47			33	20–50	0.235
Charlson's comorbidity index			0	0–1			0	0–1	0.582
WBC (n*10^3/mm^3)			13	10–16,1			14.46	11,02–17,20	0.055
Hb (g/dL)			14.1	13–14,9			14.1	13,5–15,3	0.157
CRP (mg/dL)			3.17	0,9–8,37			3.94	1,18–11,7	0.109
CT scan	76	19.3			35	36.08			< 0,001
SARS-CoV2 infection	0	0			2	1.78			

**Fig. 2:**
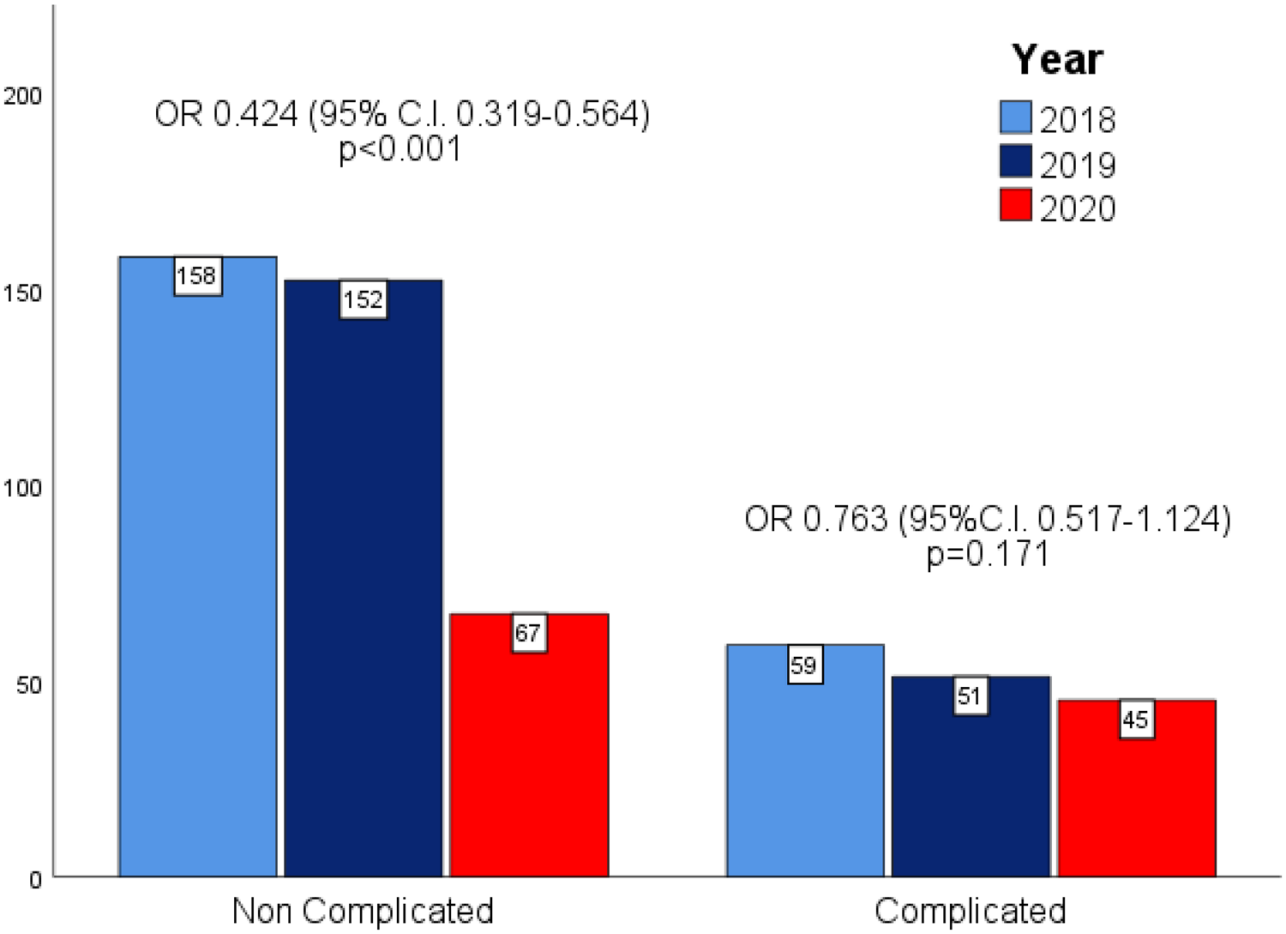
Number of non-complicated and complicated acute appendicitis during the study periods

Figure 2 shows the number of patients who were admitted for acute appendicitis during the study and control periods. The number of complicated acute appendicitis did not change (45 cases in the study period vs. a mean of 55 cases per year in the control period, − 18%, OR 0.763 95% CI 0.517–1.124 *p* = 0.1719). On the contrary, the number of non-complicated acute appendicitis significantly dropped in the study period (67 cases vs. a mean of 155 cases per year in the control period, − 56%, OR 0.424 95% CI 0.319–0.564 *p* < 0.001).

Table 2 reports operative variables and short-term postoperative outcomes in the two groups. A reduction in the negative appendectomy rate was observed (6.10% vs. 17.30%, *p* = 0.006). Morbidity and postoperative length of stay were similar in the study and control groups; no hospital readmissions were recorded.

**Table 2:** Operative variables and postoperative outcome

	Control period (2018–2019)				Study period (2020)				
	420				112				
	*N*	%	Median	IQR	*N*	%	Median	IQR	*p* value
Complicated appendicitis	110	26.19			45	40.17			0.004
Abscess	67	16.00			26	23.21			0.072
Perforation	50	11.90			19	16.96			0.157
Diffuse peritonitis	32	7.61			14	12.50			0.102
Treatment									
Non-operative	51	12.10			13	11.60			0.434
Operative	369	87.90			99	88.40			
Time to surgery (hours)			12	6–22			12	6–22	0.957
Surgical Technique									
Laparoscopy	310	84.00			90	90.90			0.1
Open	59	16.00			9	9.10			
Duration of surgery (min)			60	45–75			65	50–88	0.023
Negative appendectomy	64	17.34			6	6.10			0.006
Morbidity	33	7.90			10	8.90			0.746
Complications (grade)									
0	359	91.60			86	89.60			0.272
I	16	4.10			3	3.10			
II	10	2.60			2	2.10			
III	6	1.50			5	5.20			
IV	0	0.00			0	0.00			
V	1	0.30			0	0.00			
Length of stay			3	2–5			3	2–5	0.405
Readmission	0	0.00			0	0.00			

There were two patients with acute appendicitis and the concomitant SARS-CoV2 infection. One resulted positive at the admission screening without respiratory symptoms, and she was treated conservatively with an uneventful course. The other underwent laparoscopic appendectomy for a complicated acute appendicitis and developed acute respiratory failure after surgery when he was found positive to COVID. He needed ventilatory support, and he was safely discharged home after 16 days.

**Fig. 3:**
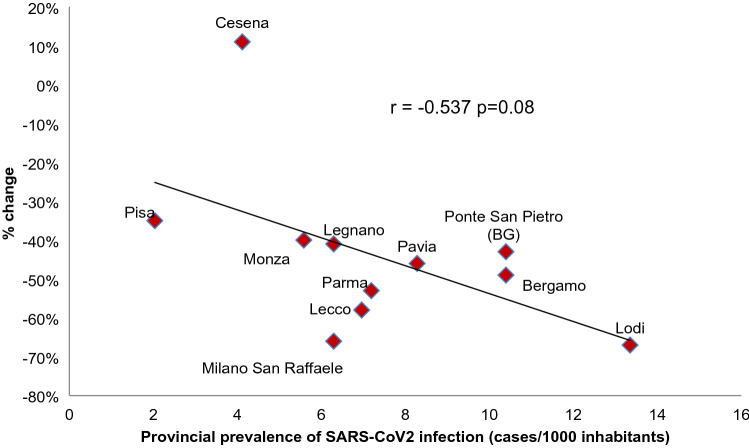
Linear correlation between prevalence of SARS-CoV2 infection and % change in hospital admission due to acute appendicitis

Figure 3 depicts the linear correlation between the prevalence of SARS-CoV2 infection for each province and the percent change of hospital admissions for acute appendicitis. The Pearson linear correlation coefficient was − 0.537 *p* = 0.08.

## Discussion

The present study shows that the lockdown due to pandemic in Northern Italy was associated with a significant reduction of hospital admission for acute appendicitis, particularly non-complicated ones. A significant reduction of the negative appendectomy rate was also found.

The reduction of both elective surgery and surgical emergencies during pandemic has been reported worldwide. An Italian multicentre study reported a 45% reduction of hospital admission due to surgical emergencies during the lockdown period, while an Israeli study observed a 32% decrease in emergency department admission for surgical complaints in the same period [19, 24]. Several studies reported a sharp decrease in patients who underwent appendectomy with a higher proportion of complicated acute appendicitis [25–28]. Maneck et al. found a significant decrease in non-complicated acute appendicitis, while the number of patients with complicated acute appendicitis remained stable [29].

The important reduction of hospitalization for acute appendicitis raises several interesting considerations. Hospitals were reorganized with the main focus to face the pandemic [30]. People considered emergency departments more as dangerous places plenty of SARS-CoV2-infected subjects than care places. Therefore, several patients with mild symptoms were probably treated conservatively at home by general practitioners. Our data suggest a linear relationship between the burden of SARS-CoV2 infections and reducing patients admitted to the hospital. The higher the prevalence of viral infection, the greater the reduction of emergency admission for acute appendicitis.

The reduced admission of patients with mild acute appendicitis was associated with significantly reducing the negative appendectomy rate. Some interesting speculations could be made about the complex and challenging relationship between health demand and health care availability. The spontaneous reduction of patients who moved to emergency departments for right lower quadrant pain might have facilitated surgeons in excluding patients with no acute appendicitis, as demonstrated by the dramatic decrease in the negative appendectomy rate.

Two kinds of acute appendicitis can be identified: the mild one with rare evolution toward abscess, perforation, or peritonitis could benefit from a non-operative treatment, whereas the complicated one urgently needs surgery. In the present study, the number of patients with complicated acute appendicitis remained stable when comparing the two study periods. Differently, patients with mild acute inflammation dropped during the pandemic period. Assuming that neither the SARS-CoV2 infection *per sé* nor the changes in lifestyle due to the lockdown can prevent acute appendicitis, a possible interpretation of our findings is that many patients with mild symptoms could have received a non-operative out-hospital treatment and that this approach was safe and effective with no significant increase in the number of complicated acute appendicitis. Unfortunately, if this was related to spontaneous resolution or outpatient antibiotic treatment is unknown.

A recent survey reported a trend to manage mild acute appendicitis with in-hospital non-operative treatment [31]. Our data demonstrated that both the rate of non-operative treatment and the proportion of patients treated with minimally invasive surgery were similar comparing the two study periods.

The high rate of negative appendectomy, similar to the literature rate, confirmed that the clinical diagnosis of acute appendicitis is sometimes challenging. To reduce futile surgery is necessary to improve the diagnostic process to identify patients with complicated acute appendicitis who need upfront surgery and those with non-complicated acute appendicitis, which can be treated conservatively. The increased proportion of CT scan could be a possible reason for reducing the negative appendectomy rate, providing a more accurate patients' selection.

The present study is limited by its retrospective design that could not demonstrate any causative effect; however, it includes a large number of patients with the same observed trend among all the participant hospitals. Further population studies are needed to confirm these hypotheses. Data about non-operative treatment at home also lack with no possibility to collect them.

In conclusion, the present study found a significant decrease in both admissions for non-complicated acute appendicitis and the negative appendectomy rate during the pandemic period. Conversely, admissions for complicated acute appendicitis did not change.

## Data Availability

Data are available under request to the corresponding author.
