# Correction to: The decrease of non-complicated acute appendicitis and the negative appendectomy rate during pandemic

**DOI:** 10.1007/s00068-021-01683-3

**Published:** 2021-05-31

**Authors:** Marco Ceresoli, Federico Coccolini, Stefano Magnone, Alessandro Lucianetti, Pietro Bisagni, Teodora Armao, Luca Ansaloni, Mauro Zago, Massimo Chiarugi, Fausto Catena, Marco Braga, the Appendicitis-COVID study group, Marco Nizzardo, Luca Nespoli, Luca Fattori, Luca Degrate, Stefano Perrone, Marco Cereda, Michele Pisano, Elia Poiasina, Paolo Bertoli, Michele Ballabio, Stefano Braga, Giorgio Graziano, Dario Tartaglia, Francesco Arces, Marco Mariani, Fulvio Tagliabue, Gennaro Perrone, Alfredo Annicchiarico, Mario Giuffrida, Giovanni Ferrari, Antonio Benedetti, Niccolò Allievi, Michele Ciocca, Enrico Pinotti, Mauro Montuori, Michele Carlucci, Valentina Tomajer, Paola Fugazzola

**Affiliations:** 1 General and Emergency Surgery Dept, School of Medicine and Surgery, Milano-Bicocca University, Via Pergolesi 33, 20900 Monza, Italy; 2 Emergency Surgery and Trauma Center Dept, Pisa University Hospital, Pisa, Italy; 3 General and Emergency Surgery Dept, ASST Papa Giovanni XXIII, Bergamo, Italy; 4 General and Emergency Surgery Dept, ASST Lodi, Lodi, Italy; 5 General and Emergency Surgery Dept, IRCCS San Matteo, University of Pavia, Pavia, Italy; 6 Robotic and Emergency Surgery Dept, ASST Lecco, Ospedale Manzoni, Lecco, Italy; 7 Emergency Surgery Dept, Parma University Hospital, Parma, Italy

## Correction to: European Journal of Trauma and Emergency Surgery 10.1007/s00068-021-01663-7

The original version of this article unfortunately contained a mistake.

The Acknowledgement with the members of the Appendicitis-COVID study group is missing. The correct version of is given below.

**Acknowledgements**

Members of the Appendicitis-COVID study group:

Monza: Marco Nizzardo, Luca Nespoli, Luca Fattori, Luca Degrate, Stefano Perrone, Marco Cereda

Bergamo: Michele Pisano, Elia Poiasina, Paolo Bertoli

Lodi: Michele Ballabio, Stefano Braga

Pavia: Giorgio Graziano

Pisa: DarioTartaglia, Francesco Arces

Lecco: Marco Mariani, Fulvio Tagliabue

Parma: Gennaro Perrone, Alfredo Annicchiarico, Mario Giuffrida

Legnano: Giovanni Ferrari, Antonio Benedetti, Niccolò Allievi

Ponte San Pietro: Michele Ciocca, Enrico Pinotti, Mauro Montuori

San Raffaele: Michele Carlucci, Valentina Tomajer

Cesena: Paola Fugazzola

The original article has been corrected.

